# Comparative assessment of “plaque/media” change on three modalities of IVUS immediately after implantation of either everolimus-eluting bioresorbable vascular scaffold or everolimus-eluting metallic stent in Absorb II study

**DOI:** 10.1007/s10554-016-1033-7

**Published:** 2016-12-23

**Authors:** Yaping Zeng, Rafael Cavalcante, Erhan Tenekecioglu, Pannipa Suwannasom, Yohei Sotomi, Carlos Collet, Mahammad Abdelghani, Hans Jonker, Franck Digne, Dieter Horstkotte, Manfred Zehender, Ciro Indolfi, Francesco Saia, Rosario Fiorilli, Bernard Chevalier, Leonardo Bolognese, Javier Goicolea, Shaoping Nie, Yoshinobu Onuma, Patrick W. Serruys, On behalf of the investigators of Absorb II study

**Affiliations:** 1 ThoraxCentre, Erasmus Medical Center, Rotterdam, The Netherlands; 2 The Emergency & Critical Care Center of Beijing Anzhen Hospital, Capital Medical University, Beijing, People’s Republic of China; 3 Academic Medical Center, Amsterdam, The Netherlands; 4 Cardialysis BV, Rotterdam, The Netherlands; 5 The Cardiology Department, Centre Cardiologique du Nord, Saint Denis, France; 6 The Department of Cardiology, Heart and Diabetes Center North Rhine-Westphalia, Ruhr University Bochum, Bad Oeynhausen, Germany; 7 Department of Cardiology and Angiology, Heart Center University of Freiburg, Freiburg, Germany; 8 Division of Cardiology, Department of Medical and Surgical Sciences, University Magna Graecia, Campus di Germaneto, Catanzaro, Italy; 9 Cardiology Institute, Policlinico S. Orsola-Malpighi, University of Bologna, Bologna, Italy; 10 Division of Cardiology, San Camillo Hospital, Rome, Italy; 11 Institute Jacques Cartier, Massy, France; 12 Cardiovascular and Neurological Department, Azienda Ospedaliera Arezzo, Arezzo, Italy; 13 Department of Interventional Cardiology, Puerta de Hierro University Hospital, Madrid, Spain; 14 International Centre for Circulatory Health, Imperial College London, South Kensington Campus, London, SW7 2AZ UK; 15 Westblaak 98, 3012KM Rotterdam, The Netherlands

**Keywords:** IVUS, Plaque/media volume, Absorb II, Bioresorbable vascular scaffold

## Abstract

The purpose of the study to assess the comparability of immediate changes in plaque/media volume (PV) on three modalities of intravascular ultrasound (IVUS) after implantation of either bioresorbable vascular scaffold (BVS) or everolimus-eluting metallic stent (EES) in Absorb II Study. The two devices have different device volume and ultrasound backscattering that may interfere with the “plaque/media” assessed by three modalities on IVUS: grayscale, backscattering of radiofrequency and brightness function. In a multicenter randomized controlled trial, 501 patients with stable or unstable angina underwent documentary IVUS pre- and post- implantation. The change in plaque/media volume (PV) was categorized into three groups according to the relative PV change in device segment: PV “increased” >+5% (PVI), PV unchanged ±5% (PVU), and PV decreased <−5% (PVD). The change in PV was re-evaluated three times: after subtraction of theoretical device volume, after analysis of echogenicity based on brightness function. In 449 patients, 483 lesions were analyzed pre- and post-implantation. “PVI” was more frequently observed in BVS (53.8%) than EES group (39.4%), p = 0.006. After subtraction of the theoretical device volume, the frequency of “PVI” decreased in both BVS (36.2%) and EES (32.1%) groups and became comparable (p = 0.581). In addition, the percentage of “PVI” was further reduced in both device groups after correction for either radiofrequency backscattering (BVS 34.4% vs. EES 22.6%) or echogenicity (BVS 25.2% vs. EES 9.7%). PV change in device segment was differently affected by BVS and EES devices implantation due to their differences in device volume and ultrasound backscattering. It implies that the lumen volume was also artifactually affected by the type of device implanted. Comparative IVUS assessment of lumen and plaque/media volume changes following implantation of BVS and EES requires specific methodological adjustment.

## Introduction

IVUS has been applied universally to understand coronary atherosclerosis, to recognize high-risk plaque features and to evaluate stent placement [1, 2]. IVUS measurements are performed at the leading edge of the ultrasonic interface, as the differential echogenic signal across the leading edge is more obvious and reproducible [3, 4]. After implantation of a metallic stent, the lumen contour is conventionally delineated along the endoluminal leading edge of the device, as it is difficult to distinguish the trailing edge due to the high ultrasonic backscattering of the metal. Therefore, the interference of the metal on the ultrasound renders the assessment of luminal and plaque/media measurements less accurate [3, 5].

By nature, polymeric material made of polylactide has a different ultrasonic interference on luminal and plaque/media measurements. In addition, differences in strut thickness, footprint area, device size, volume, and mass between metallic stent and polymeric scaffold may also have specific impact on luminal and plaque/media measurements [3, 6].

Whether different device volume and ultrasound backscattering in the two devices have differential impact on the grayscale-IVUS assessment of lumen and plaque/media remains to be investigated.

In the ABSORB II randomized trial (Clinical Trials. gov NCT01425281), pre- and post-procedural documentary IVUS imaging were mandatory. The aim of this study is to evaluate the difference in plaque/media volume change immediately after implantation of either bioresorbable vascular scaffold (BVS) or everolimus-eluting metallic stent (EES).

## Methods

### Study design and population

The ABSORB II trial is a prospective, single-blind, multicenter clinical trial that randomized patients to PCI with either BVS or EES in a 2:1 fashion. The trial design has been described in detail previously [6, 7]. In brief, the protocol of the trial allowed the treatment of up to two de-novo native coronary artery lesions, all with an angiographic maximal luminal diameter between 2.25 and 3.8 mm as estimated by online quantitative coronary angiography (QCA) and a lesion length of ≤48 mm. All patients underwent documentary grayscale-IVUS assessment before and after device implantation [6, 7].

### Study devices

The second-generation Absorb BVS is arranged as in-phase zigzag hoops linked together by three longitudinal bridges. Detail information of the device has been published [7]. The control device, EES, share its same basic MULTI-LINK design, and both devices are similar in terms of drug type, drug dose density, and elution profile. The metallic platform is made of cobalt chromium alloy. However, there are differences between the two devices in terms of device volume, mass, footprint, thickness properties, e.g.: (1) physically the strut thickness of the scaffold is approximately 150 µm, whereas the strut thickness of EES is 81 µm [7]; (2) the device volume of the BVS is approximately three times larger than the EES device for same nominal device size (Table 3).

### IVUS image acquisition, analysis and definitions

IVUS was mandatory pre- and post-implantation. In brief, IVUS data was acquired with a 3.2-French, 45-MHz rotational IVUS catheter (Revolution® 45 MHz; Volcano Corporation, Rancho Cordova, CA). IVUS data was acquired with a pullback speed of 0.5 mm/s and a frame speed of 30 frames/s.

Quantitative IVUS analysis was performed using a dedicated software (QCU-CMS-Research software v4.69, Medis, Leiden, The Netherlands) as described previously [3, 5]. The region of interest (ROI) was defined as in-device segment. The pre-procedure device segment was co-localized and matched with post-implantation using identical landmarks such as side branches, calcification, pericardium, vein, and plaque shape [3, 5]. All pullbacks were analyzed off-line by an independent core laboratory (Cardialysis BV, Rotterdam, The Netherlands).

The absolute change in IVUS measurements between pre- and post-implantation was calculated as post-implantation values minus pre-procedure values. Plaque volume (PV) was normalized by segment length when comparison was performed between BVS and EES groups. Normalized plaque/media volume = [(total vessel volume − total lumen volume)]/segment length × mean segment length in the whole population [8–11]. Relative change was calculated as absolute change divided by pre-procedure values. In previous studies, the standard deviation of intra and inter-observer variability of the IVUS measurements of plaque/media has been reported to be between 2.9 and 5%. To account for this variability with a conservative margin, relative increase or decrease in plaque/media volume was considered when a change larger than ±5.0% was observed [12–14]. The PV change was, thus, categorized into three groups: PV “increased” (PVI), PV unchanged (PVU) and PV decreased (PVD).

### VH-IVUS analysis

On VH-IVUS analysis, four major tissue components (fibrous: green; fibrofatty: light green; dense calcium, DC: white; and necrotic core: red) were characterized and compared between the two groups [15]. Post implantation pseudo “DC” was defined as confluent, non-interrupted white color surrounded by red color, located near the lumen contour not present at preprocedure images (Fig. 3, panel c). Dense calcium located behind the struts and separated from the struts was considered as real DC [16].

### Automatic quantitative echogenicity (EG) analysis on IVUS

The principle of EG has been previously described [17, 18]. EG aims to classify the vessel wall components located between the luminal boundary and the EEM into categories based on their grey-scale intensity level in B-mode IVUS images. Five tissue types were quantified: calcified, hyperechogenic, upperechogenic, hypoechogenic, and unknown [19].

### Re-evaluation of the plaque/media volume

Three types of PV re-evaluations were performed to correct for the artificial overestimation of the plaque/media volume on IVUS related to the strut volume counted as plaque/media and backscattering: first, we subtracted from the PV post-implantation the theoretical device volume disclosed by the manufacturer; second, we subtracted the post-procedural “increase in volume of VH pseudo “DC” (∆DC = post − pre)”, generated by the radiofrequency backscattering of polymeric or metallic struts, that has been considered as a surrogate assessment of the struts presence [20]; third, we subtracted the increase in volume of Upper + Hyper echogenicity (∆ Upper + Hyper EG = post − pre), which can be used as another surrogate for the device presence [19]. (Fig. 3).

### Statistical analysis

All analyses were performed on the intention-to-treat basis, using all patients randomized in the study, regardless of the treatment actually received. The Kolmogorov–Smirnov test was used to evaluate the normality assumption of all continuous variables. All continuous variables were presented as mean ± standard deviation. One-way ANOVA with Tukey’s post-hoc test or Kruskal–Wallis test were used for comparisons of continuous variables. The counts of relative change in PV were summarized and tabulated according to the frequency; Chi square test was used for categorical variables. All statistical tests were performed with SPSS version 22 (IBM Corp, Armonk, NY). A 2-sided p value of <0.05 was considered to indicate statistical significance.

## Results

### Patient and lesion characteristics

**Fig. 1:**
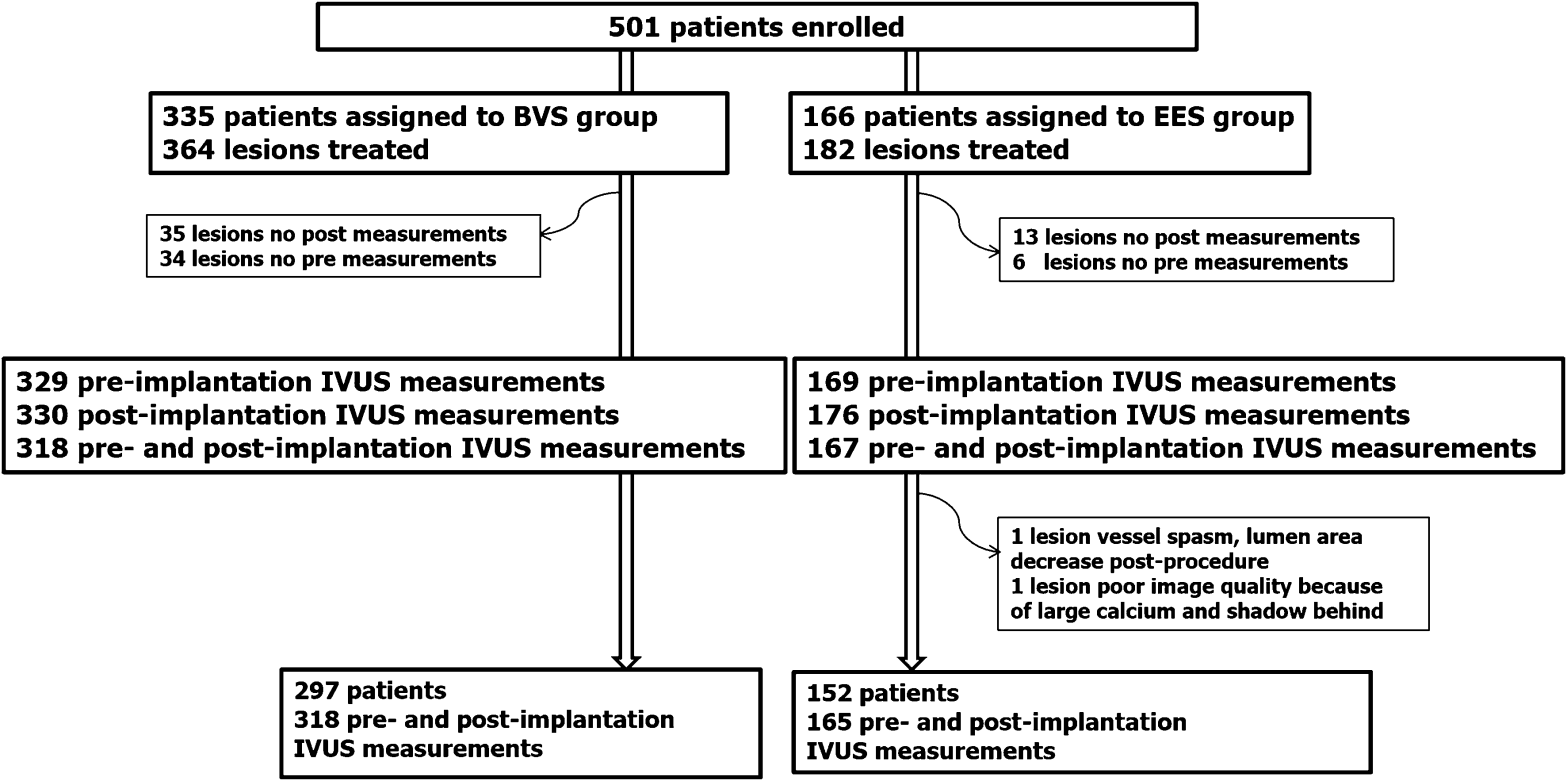
Study flow chart. *BVS* bioresorbable vascular scaffold, *EES* everolimus-eluting stent, *IVUS* intravascular ultrasound

In the ABSORB II trial, a total of 501 patients were included (BVS 335 patients, 67%; EES 166 patients, 33%). Grayscale-IVUS imaging pre- and post-implantation was available in 449 patients with 483 lesions with 318 (66%) lesions being treated with BVS and 165 (44%) lesions treated with EES (Fig. 1). This comprises the population of the present study. Overall, mean age was 63 ± 10 years, 76.6% were male and 22.9% were diabetics. Unstable angina according to the Braunwald classification was the clinical presentation in 20.7% of cases. The study arms were well balanced with regard to baseline clinical characteristics (Table 1).

**Table 1:** Baseline patient characteristics in Absorb II study (449 patients, 483 lesions)

449 patients	BVS (297 patients)	EES (152 patients)	p value
Age, years	63.6 ± 10.1	63.6 ± 9.9	0.987
Male, n (%)	223 (75.1%)	121 (79.6%)	0.346
Hypertension, n (%)	191 (64.3%)	102 (67.1%)	0.804
Hypercholesterolemia, n (%)	208 (70%)	112 (73.7%)	0.328
Diabetes mellitus, n (%)	65 (21.9%)	38 (25%)	0.458
Myocardial infarction history, n (%)	80 (26.9%)	46 (30.3%)	0.596
Cardiac Intervention history, n (%)	102 (34.3%)	54 (35.5%)	0.834
Current smokers, n (%)	76 (25.6%)	34 (22.4%)	0.118
Family history of CHD, n (%)	99 (33.3%)	56 (36.8%)	0.76
Clinic presentation, n (%)			0.629
Stable angina	192 (64.6%)	98 (64.5%)	
Unstable angina	60 (20.2%)	33 (21.7%)	
Silent ischemia	35 (11.8%)	19 (12.5%)	

483 lesions	BVS (318 lesions)	EES (165 lesions)	p value
Target vessel			0.166
Left anterior descending, n (%)	89 (28%)	80 (48.5%)	
Left circumflex, n (%)	148 (46.5%)	34 (20.6%)	
Right coronary artery, n (%)	81 (25.5%)	51 (30.9%)	
AHA/ACC lesion classification			0.197
A, n (%)	4 (1.3%)	1 (0.6%)	
B1, n (%)	184 (57.9%)	83 (50.3%)	
Type B2/C lesion, n (%)	130 (40.9%)	81 (49.1%)	

Data are shown in n (%) or mean ± SD

*BVS* bioresorbable vascular scaffold, *EES* everolimus-eluting metallic stent, *CHD* coronary heart disease

### IVUS measurements pre- and post-implantation

**Fig. 2:**
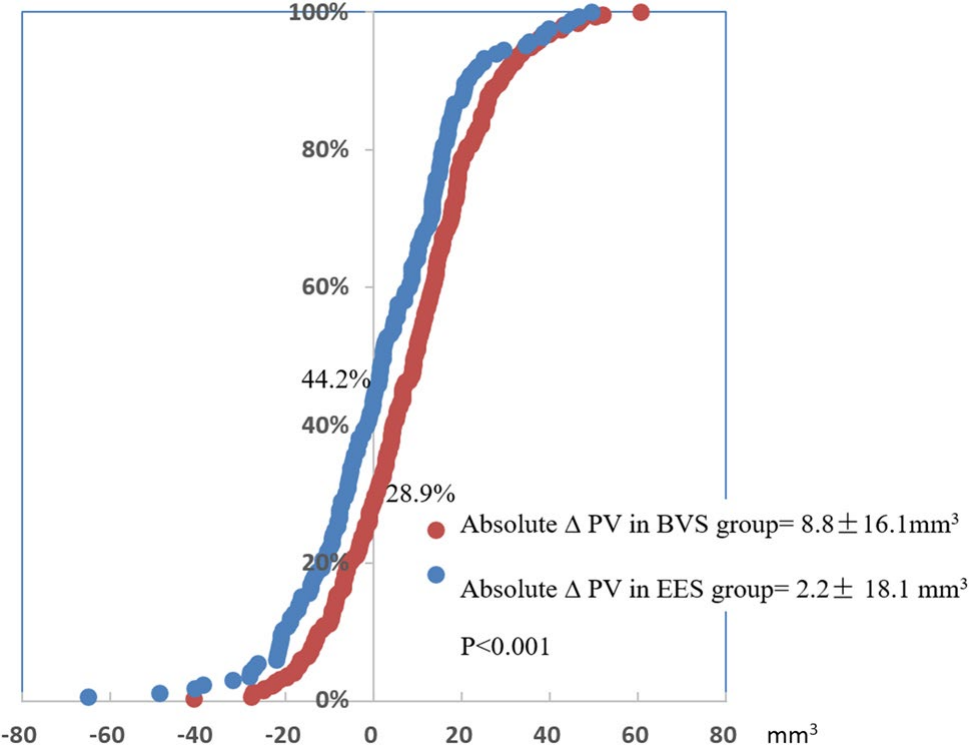
Cumulative frequency distribution curves of absolute ΔPV (mm^3^) pre- and post-implantation in device segment in BVS and EES groups. In BVS group, more than 70% of the lesions showed PV “increase” post-implantation; in EES group, approximately 50% of the lesions showed PV “increase” post-implantation. *BVS* bioresorbable vascular scaffold, *EES* everolimus-eluting stent, *PV* plaque/media volume

On pre-implantation IVUS, average plaque/media volume was smaller in the BVS group (154.7 ± 58.7 mm^3^ vs. 168.8 ± 62.6 mm^3^; p = 0.015). Average PV showed a higher increase in the BVS group (ΔPV 8.8 ± 16.1 mm^3^ vs. 2.2 ± 18.1 mm^3^; p < 0.001) and at post-implantation, there was no difference in average PV in both arms (BVS 163.5 ± 56.9 mm^3^ vs. EES 170.9 ± 56.4 mm^3^; p = 0.172). Increase in PV was observed in BVS in 53.8% of cases and in EES in 39.4% of cases, p = 0.006 (Fig. 2).

### PV changes after correction for device volume

#### Subtraction of theoretical device volume

Average theoretical device volume was 9.4 ± 1.0 mm^3^ in the BVS group and 2.8 ± 1.0 mm^3^ in the EES group (p < 0.001). After subtraction of theoretical device volume, delta PV was no longer different between the two study arm (BVS ∆PV 0.6 ± 14.6 mm^3^ vs. EES ∆ PV 0.5 ± 15.9 mm^3^; p = 0.963) (Table 2). However, the frequency of this “increase” in average PV was reduced to 36.2% in the BVS arm and 32.1% in the EES arm; p = 0.581.

**Table 2:** IVUS measurements pre- and post-implantation in Absorb II study (449 patients, 483 lesions)

	BVS 318 lesions	EES 165 lesions	p value
Plaque volume			
Pre-procedure (mm^3^)	154.7 ± 58.7	168.8 ± 62.6	0.015
Post-procedure (mm^3^)	163.5 ± 56.9	170.9 ± 56.4	0.172
Δ post-pre procedure (mm^3^)	8.8 ± 16.1	2.2 ± 18.1	<0.001
Relative Δ post-pre procedure (%)	7.7 ± 12.9	3.6 ± 14.0	0.001
“PVI”, n (%)	171, 53.8%	65, 39.4%	0.006
Vessel volume			
Pre-procedure (mm^3^)	262.8 ± 78	281.1 ± 77.9	0.015
Post-procedure (mm^3^)	302 ± 82.1	328.1 ± 82.1	0.001
Δ post-pre procedure (mm^3^)	39.2 ± 20.7	47 ± 20.6	<0.001
Relative Δ post-pre procedure (%)	16.1 ± 9.7	17.8 ± 8.8	0.056
Mean lumen volume			
Pre-procedure (mm^3^)	108.1 ± 31.1	112.3 ± 32	0.162
Post-procedure (mm^3^)	138.5 ± 33.3	157.2 ± 36.4	<0.001
Δ post-pre procedure (mm^3^)	30.4 ± 19.7	44.8 ± 22.8	<0.001
Relative Δ post-pre procedure (%)	31.5 ± 22.8	44.2 ± 27.3	<0.001
Plaque burden (%)	57.1 ± 8.6	58.6 ± 8.8	0.077

Data are shown in n (%) or mean ± SD

*BVS* bioresorbable vascular scaffold, *EES* everolimus-eluting metallic stent, *PVI* plaque volume increase

#### Subtraction of pseudo dense calcium on Virtual Histology-IVUS

Average pseudo “dense calcium” volume was comparable in the two groups (BVS 8.3 ± 6.0 mm^3^ vs. EES 8.0 ± 5.6 mm^3^; p = 0.605). After subtraction of pseudo “dense calcium” volume, delta PV was different between the two study arms (BVS ∆PV 0.65 ± 16.8 mm^3^ vs. EES ∆PV −5.7 ± 18.7 mm^3^; p < 0.001). However, the frequency of “increase” in average PV was reduced to 34.4% in the BVS and 22.6% in the EES arms; p = 0.004.

#### Subtraction of pseudo “Upper + Hyper echogenicity”

Average pseudo “Upper + Hyper echogenicity” volume in BVS group (12.5 ± 12.6 mm^3^) was significantly less than in EES group (18.4 ± 10.5 mm^3^), p < 0.001.

After subtraction of pseudo “Upper + Hyper echogenicity” volume, delta PV was different between the two study arms (BVS ∆PV −3.7 ± 18.8 mm^3^ vs. EES ∆PV −16.3 ± 21.4 mm^3^; p < 0.001). The frequency of “increase” in average PV was drastically less than before subtraction in both device arms (BVS 25.2% vs. EES 9.7%; p < 0001) (Table 3).

**Table 3:** IVUS measurements of plaque volume in device segment pre- and post-implantation after re-evaluations in Absorb II study (449 patients, 483 lesions)

	BVS 318 lesions	EES 165 lesions	p value
Subtraction of theoretic device volume			
Theoretic device volume (mm^3^)	9.4 ± 1.0	2.8 ± 1.0	<0.001
Post PV subtraction of the theoretic device volume (mm^3^)	154.0 ± 56.6	168.2 ± 56.2	0.009
∆PV post-pre after subtraction of the theoretic device volume (mm^3^)	0.60 ± 14.6	0.53 ± 15.9	0.963
Relative ∆PV post-pre after subtraction of the theoretic device volume (%)	1.5 ± 15.2%	1.3 ± 16.2%	0.904
p Value pre vs. post subtraction of the theoretic device volume	0.445	0.665	
Pseudo “PVI”, lesions, n(%)	115, 36.2%	53,32.1%	0.581
Subtraction of pseudo “DC” on VH-IVUS*			
Pseudo “DC” on VH (mm^3^)	8.3 ± 6.0	8.0 ± 5.6	0.605
Post PV subtraction of the pseudo “DC” (mm^3^)	155.7 ± 57.7	162.9 ± 57.7	0.209
∆PV post-pre after subtraction of the pseudo “DC” (mm^3^)	0.65 ± 16.8	−5.7 ± 18.7	<0.001
Relative ∆PV post-pre after subtraction of the pseudo “DC” (%)	1.7 ± 13.3	−2.0 ± 14.2	0.007
p Value pre vs. post after subtraction pseudo “DC”	0.509	<0.001	
Pseudo “PVI”, n (%)	101, 34.4%	36, 22.6%	0.004
Subtraction of pseudo “Upper + Hyper EG” on echogenicity			
Pseudo “Upper + Hyper” volume (mm^3^)	12.5 ± 12.6	18.4 ± 10.5	<0.001
Post PV subtraction of pseudo “Upper + Hyper” volume (mm^3^)	151.0 ± 53.5	152.5 ± 53.6	0.769
∆PV post-pre after subtraction of pseudo “Upper + Hyper” (mm^3^)	−3.7 ± 18.8	−16.3 ± 21.4	<0.001
Relative ∆PV post-pre after subtraction of pseudo “Upper + Hyper” (%)	−0.77 ± 13.7	−8.3 ± 12.5	<0.001
p Value pre vs. post after subtraction of pseudo “Upper + Hyper”	<0.001	<0.001	
Pseudo “PVI”, n (%)	80, 25.2%	16, 9.7%	<0.001

Data are shown in n or mean ± SD. The volume value was normalized by the mean of segment length in the all population

*BVS* bioresorbable vascular scaffold, *EES* everolimus-eluting metallic stent, *PV* plaque volume, *DC* dense calcium, *VH*-*IVUS* virtual histology-IVUS, *Pseudo* “*PVI*” pseudo “plaque volume increase”

*VH-IVUS analysis are available in 453 lesions, BVS = 294 lesions, EES = 159 lesions

## Discussion

In the present study, we evaluated how the plaque/media volume changes following implantation of either BVS or EES. The major findings of this study are the following: (1) acute plaque/media volume change after implantation was differently influenced by the device: plaque/media volume overestimation was more frequently observed in BVS than in EES groups, due to different device volumes and ultrasound backscattering. (2) re-evaluations could be performed to minimize the overestimation of plaque/media volume.

### Plaque/media volume overestimation after implantation

Before correction for the device volume and backscattering, plaque/media volume “increase” was observed in both BVS and EES arms. The possible explanation is that IVUS lacks the necessary resolution to detect the true lumen border by differentiating the abluminal boundaries of device struts. Therefore, device volumes and their backscattering are artifactually defined as “plaque/media volume” on grayscale-IVUS (Fig. 4a, a′) [5].

This artificial plaque/media volume overestimation was more frequently observed in BVS group than in EES group, due to the fact that the device volume of the BVS is more than three times larger than the EES device for same nominal device size.

### Re-evaluations were applied to minimize the plaque/media overestimation

After correction for the theoretical device volume, there was no longer significant difference in ∆PV between BVS and EES groups. They presented comparable percentage of plaque/media overestimation. However, even after correction for the theoretical device volume, PV “increase” was still observed in more than 1/3 of the lesions in both device arms, due to device backscattering on IVUS.

**Fig. 3:**
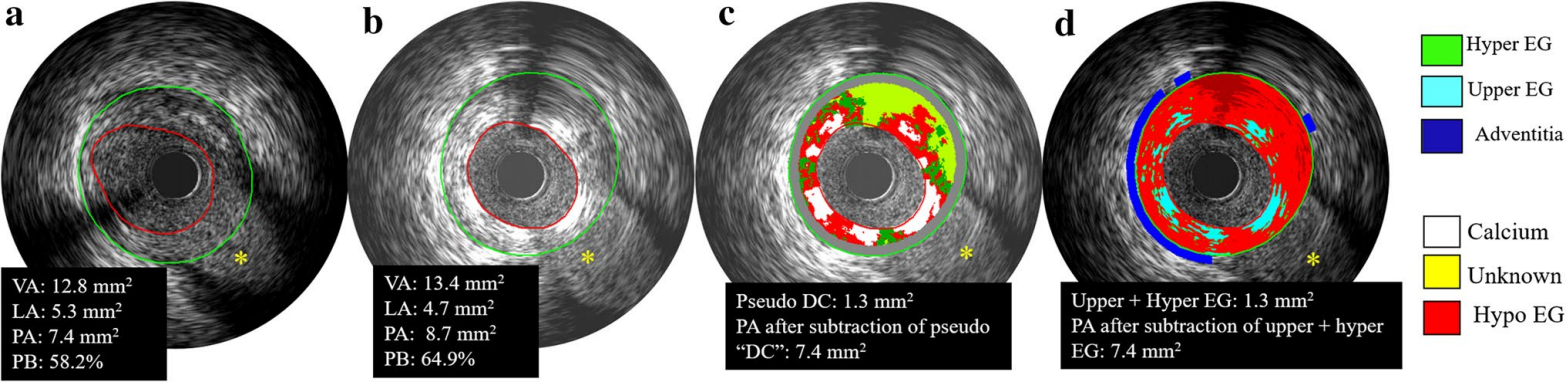
Examples showing the overestimation and re-evaluations of plaque/media volume after implantation. The PA was over estimated from 7.4 to 8.7 mm^2^, as well as the PB (Pre: 58.2% vs. 64.9%) (*Panels* **a, b**). As IVUS lacks the necessary resolution to detect the true lumen border by differentiating the abluminal boundaries of device struts. The lumen contour is conventionally delineated along the endoluminal leading edge of the device. Therefore, device volumes and their backscattering are artifactually defined as “plaque/media volume” and was overestimated on grayscale-IVUS. PA was re-evaluated after subtraction of the pseudo “DC” on VH-IVUS and the Upper + Hyper EG which are surrogates for the device presence in both lesions (*Panels* **c, d**). One of the disadvantage of VH-IVUS is the default presence of medial stripe that masks and hides some of the struts in direct contact with the adventitia. Echogenicity is able to assess the plaque/media area without the hiding effect of the medial stripe of VH-IVUS. *VA* vessel area, *LA* lumen area, *PA* plaque/media area, *PB* plaque burden, *VH*-*IVUS* virtual histology-IVUS, *EG* echogenicity. *Side branches; *DC* dense calcium

Re-evaluations were further performed to subtract of the device appearance on backscattering virtual histology-IVUS, based on the fact that device struts are artifactually presented as pseudo “DC” on VH [16, 20]. After re-evaluation, the overestimation of PV was minimized in both device groups. Nevertheless, one of the disadvantages of VH is the default presence of a medial grey stripe that masks and hides some of the struts in direct contact with the adventitia affecting the results of this correction (Fig. 3).

Similarly to VH, device struts appear as pseudo “Upper + Hyper echogenicity” on echogenicity analyses, which has been previously validated [5, 19, 21, 22]. On further re-evaluation after correction for the pseudo “Upper + Hyper echogenicity”, the frequency of plaque/media volume “increase” was significantly reduced. The advantage of echogenicity is that it is able to assess the plaque/media area without the hiding effect of the medial stripe of the VH-IVUS (Fig. 3).

**Fig. 4:**
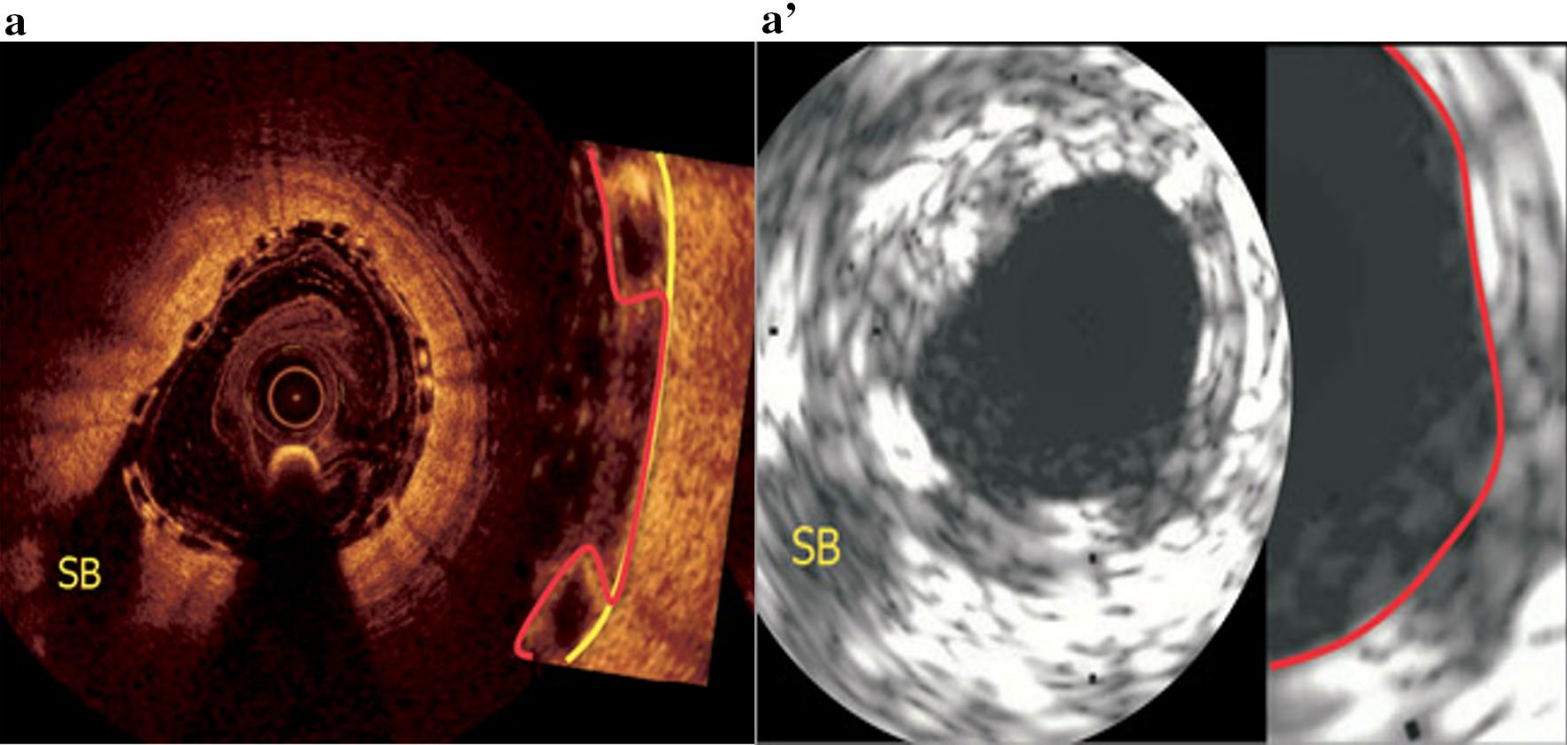
Compared to OCT, IVUS overestimates the plaque/media. *Panels* **a** and **a′** show one patient who had undergone both IVUS (20 MHz) and OCT investigation, one cross section was matched using the side branch [5]. The lumen contour on IVUS mainly relies on the highly reflective endoluminal surface of the polymeric strut and may lack the necessary imaging resolution to detect the true boundaries of the vessel wall between the protruding polymeric struts. Therefore, IVUS may underestimate the lumen area and conversely overestimates the plaque/media area. In contrast, the contour of the true flow lumen area on OCT does not include artifactually the space filled with blood flowing between the protruding polymeric struts [5, 21]. *SB* side branch Reprinted with permission from Serruys et al. [5]

Nonetheless, even after re-evaluations, PV overestimations persisted in both groups. This was due to the space between struts being still artifactually defined as “plaque/media” given the limited resolution of IVUS as mentioned above. In contrast, the contour of the true lumen area on OCT does not include artifactually the space filled with blood flowing between the protruding struts (Fig. 4) [5, 21]. Hybrid catheter co-registration of the two imaging modalities could, thus, provide a more accurate assessment of the lumen and plaque/media measurements [23]. Before hybrid are available in clinical, OCT may be an alternative, despite possibly limited imaging of larger plaque or thickened wall.

Interestingly, the correction using the theoretical device volume yielded different results when compared to the correction using VH or EG. This happened because while the theoretical device volumes of EES and BVS are significantly different (BVS is more than three times larger), this difference is reduced in the in vivo images mainly due to the relatively low resolution of IVUS. For that reason, PMV changes behave differently following correction using these different methods.

### Study strengths and limitations

Our study must be viewed in light of some limitations. The ABSORB II trial included mostly patients with less complex coronary lesions and our findings might not be extrapolated to more complex scenarios. Furthermore, we do not provide a comparison with a gold-standard method of acute plaque/media changes after BVS and EES implantation. However, such a method is still unavailable in clinical practice. Moreover, our study derives from a solid database of a large randomized controlled trial and our data was analyzed by an independent core laboratory.

## Conclusion

Acute plaque/media volume change was differently affected by BVS and EES devices implantation due to their differences in device volume and ultrasound backscattering patterns. This implies that the lumen volume may also be artificially affected by the implanted device. Comparative assessment of lumen and plaque/media change on IVUS following BVS and EES implantation requires methodological adjustment. Hybrid catheters that combine OCT and IVUS images could potentially provide a more accurate assessment of the respective mechanical performances of each device.

## Abbreviations

BVS: Bioresorbable vascular scaffold
EES: Everolimus-eluting metallic stent
QCA: Quantitative coronary angiography
VH-IVUS: Virtual histology-intravascular ultrasound
ROI: Region of interest
PV: Plaque/media volume
PVI: Plaque volume increased
PVU: Plaque volume unchanged
PVD: Plaque volume decreased
